# Duration of Postdiarrheal Enteric Pathogen Carriage in Young Children in Low-resource Settings

**DOI:** 10.1093/cid/ciaa1528

**Published:** 2020-10-09

**Authors:** Timothy L McMurry, Elizabeth T Rogawski McQuade, Jie Liu, Gagandeep Kang, Margaret N Kosek, Aldo A M Lima, Pascal O Bessong, Amidou Samie, Rashidul Haque, Estomih R Mduma, Jose Paulo Leite, Ladaporn Bodhidatta, Najeeha T Iqbal, Nicola Page, Ireen Kiwelu, Zulfiqar A Bhutta, Tahmeed Ahmed, Eric R Houpt, James A Platts-Mills

**Affiliations:** 1 Department of Public Health Sciences, University of Virginia, Charlottesville, Virginia, USA; 2 Division of Infectious Diseases and International Health, University of Virginia, Charlottesville, Virginia, USA; 3 Christian Medical College, Vellore, India; 4 Asociación Benéfica PRISMA, Iquitos, Peru; 5 Federal University of Ceara, Fortaleza, Brazil; 6 University of Venda, Thohoyandou, South Africa; 7 International Centre for Diarrhoeal Disease Research, Bangladesh, Dhaka, Bangladesh; 8 Haydom Global Health Research Centre, Haydom, Tanzania; 9 Fundação Oswaldo Cruz, Rio de Janeiro, Brazil; 10 Armed Forces Research Institute of Medical Sciences, Bangkok, Thailand; 11 Aga Khan University, Karachi, Pakistan; 12 National Institute for Communicable Diseases, Johannesburg, South Africa; 13 Kilimanjaro Clinical Research Institute, Moshi, Tanzania

**Keywords:** diarrhea, asymptomatic carriage, children, shedding, incubation period

## Abstract

**Background:**

Prolonged enteropathogen shedding after diarrhea complicates the identification of etiology in subsequent episodes and is an important driver of pathogen transmission. A standardized approach has not been applied to estimate the duration of shedding for a wide range of pathogens.

**Methods:**

We used a multisite birth cohort of children 0–24 months of age from whom diarrheal and monthly nondiarrheal stools were previously tested by quantitative polymerase chain reaction for 29 enteropathogens. We modeled the probability of detection of the etiologic pathogen before and after diarrhea using a log-normal accelerated failure time survival model and estimated the median duration of pathogen carriage as well as differences in subclinical pathogen carriage 60 days after diarrhea onset in comparison to a prediarrhea baseline.

**Results:**

We analyzed 3247 etiologic episodes of diarrhea for the 9 pathogens with the highest attributable burdens of diarrhea. The median duration of postdiarrheal carriage varied widely by pathogen, from about 1 week for rotavirus (median, 8.1 days [95% confidence interval {CI}, 6.2–9.6]) to >1 month for *Cryptosporidium* (39.5 days [95% CI, 30.6–49.0]). The largest increases in subclinical pathogen carriage before and after diarrhea were seen for *Cryptosporidium* (prevalence difference between 30 days prior and 60 days after diarrhea onset, 0.30 [95% CI, .23–.39]) and *Shigella* (prevalence difference, 0.21 [95% CI, .16–.27]).

**Conclusions:**

Postdiarrheal shedding was widely variable between pathogens, with strikingly prolonged shedding seen for *Cryptosporidium* and *Shigella*. Targeted antimicrobial therapy and vaccination for these pathogens may have a relatively large impact on transmission.

## Teaser

In a multisite birth cohort study of children 0–24 months of age and with quantitative polymerase chain reaction detection of diarrheal pathogens, we estimate the duration of postdiarrheal carriage in low-resource settings, with strikingly prolonged shedding seen for *Cryptosporidium* and *Shigella*.

The application of quantitative molecular diagnostics to studies of enteric infections in children in low-resource settings has identified a strikingly high prevalence of pathogen carriage even in the absence of clinical symptoms [1–3]. This subclinical carriage confounds the assignment of etiology when pathogens are detected from diarrheal stools. The incorporation of nondiarrheal controls in diarrhea etiology studies has helped differentiate clinically relevant detections from pathogen bystanders [1]. Subclinical enteropathogen infections have been associated with poor child growth [4], and some, but not all, subclinical enteropathogen carriage is presumed to represent postdiarrheal shedding [5]. The relative frequency of persistent postdiarrheal carriage may explain differences in the association between specific enteropathogens and growth. Finally, the duration of post- and even prediarrheal shedding is important to characterize since it can be an important driver of human-to-human pathogen transmission.

Previous estimates of the duration of shedding have come from single-pathogen studies from heterogeneous populations and study types (eg, controlled challenged models in adults) and have used a wide range of diagnostics [6–13]. The duration of postdiarrheal shedding of the most common enteric pathogens in young children in low-resource settings, using a consistent diagnostic approach, has not been defined. Precise estimation of the relative duration of postdiarrheal carriage for a range of common enteric pathogens requires large, prospective longitudinal cohorts with frequent sampling of diarrheal and nondiarrheal stools, as well as the use of uniform and sufficiently sensitive microbiologic methods. We recently estimated burdens of etiology-specific diarrhea and the association between enteric infections and linear growth using quantitative polymerase chain reaction (qPCR) in 8 diverse low-resource settings in a multisite birth cohort study, Etiology, Risk Factors, and Interactions of Enteric Infections and Malnutrition and the Consequences for Child Health and Development (MAL-ED) [2, 4]. Here, we estimate the duration of postdiarrheal carriage for the leading etiologies of diarrhea in this cohort.

## MATERIALS AND METHODS

### Clinical Data

The rationale, methodology, and principal findings of the enteric pathogen analyses from MAL-ED have been previously reported [2, 4, 14, 15]. In brief, healthy infants were enrolled from November 2009 to February 2012 in 8 diverse study sites and were followed intensively through 2 years of age, including twice-weekly home visits for surveillance of childhood illnesses and antibiotic administration. Diarrhea, defined as maternal report of ≥3 loose stools in 24 hours, or 1 stool with visible blood, was identified through home visits, and diarrheal as well monthly nondiarrheal surveillance stools were collected. Stools collected within 48 hours before or after a day of study-defined diarrhea were considered to be diarrheal stools. Diarrhea for <7 days was considered acute, for 7–13 days prolonged, and for ≥14 days persistent. Appropriate empiric treatment for childhood diarrhea was defined as caregiver-reported use of fluoroquinolone, macrolide, or cephalosporin antibiotics on at least 1 day of the diarrhea episode [16].

### Microbiologic Studies

We tested all available diarrheal and nondiarrheal stool samples from children who completed 24 months of follow-up using custom-designed TaqMan Array Cards (Thermo Fisher, Carlsbad, California) that compartmentalized qPCR assays for 29 enteropathogens [2]. All procedures, including assay validation, nucleic acid extraction, quantitative PCR setup, and quality control have been previously described [17, 18]. Raw stool aliquots were stored at −80°C before extraction. Bacteriophage MS2 was used as an external control to monitor efficiency of nucleic acid extraction and amplification. We included 1 extraction blank per batch and 1 no-template amplification control per 10 cards to exclude laboratory contamination.

### Data Analysis

We used previously developed models to assign diarrhea etiology, classifying etiologic detections of pathogens in diarrhea as those with an episode-specific attributable fraction of at least 0.5, as previously described [2]. For all analyses, we included diarrheal and nondiarrheal stools with completely valid results for all pathogens with etiologic detections in at least 1% of diarrheal episodes. Because an exact estimation of the duration of carriage after each episode of diarrhea would require a much higher intensity of sampling (eg, daily) after each episode, we instead estimated the duration of carriage by modeling the probability of pathogen re-detection after diarrhea attributable to that pathogen. To do this, we leveraged the fact that diarrhea episodes occurred at random intervals before and after scheduled collection of monthly nondiarrheal stools. First, we identified all stool samples collected within a 60-day “incubation window” prior to diarrhea onset as well as a 60-day “shedding window” after diarrhea onset, with the shedding window extended to 120 days for *Cryptosporidium* based on the observed long duration of postdiarrheal carriage for this pathogen.

The primary analysis included all diarrheal episodes except those that either (1) occurred during the shedding window of an episode attributed to the same pathogen or (2) occurred at any time after an episode attributed to the same pathogen and all stool samples collected between the episodes had the pathogen detected. These repeat episodes were excluded to remove cases that could have been due to a long, lingering infection rather than a new infection; we also assessed the impact of this choice with a sensitivity analysis that included all diarrheal episodes.

To estimate the probability of a positive stool as a function of time, we used the binary outcome model


$$\begin{array}{l} P(Y_{t}=1\;|\;X)=(1-h)\left[1-\Phi \;\left(\frac{\log t-\beta^{}X}{\sigma }\right)\right]+h. \end{array}$$ (1)

For each pathogen, this approach was used to model both the probability of a positive stool t days after diarrheal onset, and, with a separate model, the probability of a positive stool t days before onset. In the remainder of the model, $Y_{t}\;$ is a binary indicator for whether or not the pathogen was detected at t days after (or before) onset; X is a vector whose first entry is 1 (effectively an intercept) and whose remaining entries are covariates (eg, age, antibiotic treatment); β is a vector of regression coefficients; σ is a scale parameter;  Φ  is the standard normal cumulative distribution function; and h is a “floor” parameter that allows for the possibility of an ongoing background rate of carriage. Time 0 was fixed as the day of symptom onset. This model is, in essence, a log-normal accelerated failure time survival model adjusted to allow the percentage of detection to drop to a nonzero asymptote [19]. Models were fit by maximum likelihood. Finally, we used nonetiologic pathogens detected in the diarrheal stool as negative controls by using splines to model the probability of detection of these pathogens during the incubation and shedding windows.

We used model (1) to estimate the probability of pathogen detection at any time before, during, or after the episode of diarrhea, rather than to estimate time to the end of pathogen carriage, as is done with survival analysis. We chose the specific model form, rather than for example logistic regression, because it allows for a 100% positive detection rate at time 0. We also considered a modified Weibull model; however, this model, near time 0, is sensitive to the estimated shape parameter, which does not seem biologically plausible.

The median durations of pre- and postdiarrheal carriage were estimated as the time in days before and after symptom onset when there was a 50% chance of pathogen detection. Median durations of postdiarrheal carriage were also estimated for defined subgroups by including additional dichotomous covariates in the model and defining the model-predicted probabilities for each level of the covariate. The prevalence difference between 60 days after diarrhea onset and 30 days prior to diarrhea onset was calculated as the difference in the model-predicted probability of detection at those time points. Confidence intervals (CIs) were estimated by bootstrapping at the subject level. All analyses were performed using R version 3.6.0 software (R Foundation for Statistical Computing, Vienna, Austria, 2019).

## RESULTS

There were a total of 42 488 samples from 1715 children. Of these, 40 976 (96.4%) had valid qPCR results for all of the pathogens included in this analysis, including 6687 diarrheal and 34 289 monthly nondiarrheal stools. Nine pathogens were identified as etiologic in at least 1% of episodes, led by *Shigella* (736 etiologic episodes [11.0% of episodes]), rotavirus (554 [8.3%]), sapovirus (535 [8.0%]), heat-stabile toxin-producing enterotoxigenic *Escherichia coli* (ST-ETEC) (452 [6.8%]), and adenovirus 40/41 (404 [6.0%]) (Table 1). In total, there were 3551 etiologic episodes for these 9 pathogens, of which 3247 met criteria for inclusion in the analysis. Of the excluded episodes, 284 (8.0%) occurred during the shedding window of a prior etiologic episode for the same pathogen and 20 (0.6%) had no negative stool tests for the etiologic pathogen between the incident episode and a preceding episode. Between 94.4% (*Cryptosporidium*) and 68.4% (adenovirus 40/41) of included episodes were the first identified episode for that pathogen for that child. The mean child age at the time of diarrhea ranged from 11.2 months for *Campylobacter* to 16.5 months for *Shigella*. The majority of episodes had a duration of ≤7 days for all pathogens, ranging from 92.9% of rotavirus episodes to 84.8% of *Shigella* episodes (Table 1).

**Table 1.** Characteristics of Etiologic Episodes Included in the Analysis (N = 6687)

Characteristic	Adenovirus 40/41	Astrovirus	*Campylobacter jejuni/ Campylobacter coli*	*Cryptosporidium*	Norovirus GII	Rotavirus	Sapovirus	*Shigella*	ST-ETEC
Etiologic episodes	404	303	160	119	288	554	535	736	452
<60 d since a previous etiologic episode	50 (12.4)	10 (3.3)	8 (5.0)	11 (9.2)	4 (1.4)	34 (6.1)	44 (8.2)	89 (12.1)	34 (7.5)
No negative test since prior etiologic episode	0 (0.0)	1 (0.3)	1 (0.6)	0 (0.0)	0 (0.0)	0 (0.0)	2 (0.4)	10 (1.4)	6 (1.3)
Etiologic episodes included in analysis	354 (87.6)	292 (96.4)	151 (94.4)	108 (90.8)	284 (98.6)	520 (93.9)	489 (91.4)	637 (86.5)	412 (91.2)
First episode	242 (68.4)	257 (88.0)	123 (81.5)	102 (94.4)	247 (87.0)	436 (83.8)	383 (78.3)	495 (77.7)	330 (80.1)
Repeat episode	112 (31.6)	35 (12.0)	28 (18.5)	6 (5.6)	37 (13.0)	84 (16.2)	106 (21.7)	142 (22.3)	82 (19.9)
Stools available during incubation window, mean ± SD	2.3 ± 0.8	2.3 ± 0.8	2.4 ± 0.9	2.3 ± 0.8	2.3 ± 0.8	2.2 ± 0.8	2.3 ± 0.8	2.2 ± 0.8	2.2 ± 0.8
Stools available during shedding window, mean ± SD	2.2 ± 0.9	2.3 ± 0.9	2.3 ± 0.9	4.1 ± 1.8	2.2 ± 0.9	2.1 ± 0.8	2.2 ± 0.9	2.0 ± 0.8	2.1 ± 0.9
Child age at diarrhea onset, mean ± SD	11.7 ± 5.5	13.0 ± 5.8	11.2 ± 5.3	15.7 ± 5.6	12.3 ± 5.2	11.5 ± 5.7	14.1 ± 5.0	16.5 ± 5.0	14.5 ± 5.4
0–6 mo	59 (16.7)	38 (13.0)	23 (15.2)	4 (3.7)	23 (8.1)	96 (18.5)	18 (3.7)	16 (2.5)	17 (4.1)
6–12 mo	139 (39.3)	95 (32.5)	71 (47.0)	28 (25.9)	128 (45.1)	209 (40.2)	167 (34.2)	114 (17.9)	133 (32.3)
12–18 mo	109 (30.8)	92 (31.5)	40 (26.5)	34 (31.5)	88 (31.0)	144 (27.7)	190 (38.9)	230 (36.1)	134 (32.5)
18–24 mo	53 (15.0)	68 (23.3)	17 (11.3)	42 (38.9)	46 (16.2)	78 (15.0)	118 (24.1)	278 (43.6)	129 (31.3)
Episode duration, mean ± SD	4.0 ± 2.9	4.3 ± 3.4	4.2 ± 3.1	4.6 ± 3.6	4.0 ± 2.9	4.2 ± 3.0	4.2 ± 3.7	4.9 ± 4.8	4.1 ± 3.2
Acute diarrhea (<7 d)	308 (87.0)	254 (87.0)	122 (80.8)	85 (78.7)	248 (87.3)	445 (85.6)	419 (85.7)	508 (79.7)	353 (85.7)
Prolonged diarrhea (7–13 d)	41 (11.6)	29 (9.9)	26 (17.2)	19 (17.6)	34 (12.0)	67 (12.9)	59 (12.1)	104 (16.3)	50 (12.1)
Persistent diarrhea (≥14 d)	5 (1.4)	9 (3.1)	3 (2.0)	4 (3.7)	2 (0.7)	8 (1.5)	11 (2.2)	25 (3.9)	9 (2.2)
Episode treated with any antibiotic	186 (52.5)	129 (44.2)	54 (35.8)	50 (46.3)	124 (43.7)	270 (51.9)	211 (43.1)	394 (61.9)	197 (47.8)
Episode treated with appropriate empiric antibiotic	140 (39.5)	71 (24.3)	30 (19.9)	25 (23.1)	53 (18.7)	170 (32.7)	113 (23.1)	260 (40.8)	125 (30.3)
Treated with a macrolide	102 (28.8)	38 (13.0)	23 (15.2)	13 (12.0)	39 (13.7)	96 (18.5)	68 (13.9)	136 (21.4)	79 (19.2)
Treated with a fluoroquinolone	36 (10.2)	13 (4.5)	2 (1.3)	8 (7.4)	12 (4.2)	50 (9.6)	33 (6.7)	91 (14.3)	39 (9.5)
Treated with a cephalosporin	16 (4.5)	26 (8.9)	6 (4.0)	5 (4.6)	5 (1.8)	34 (6.5)	22 (4.5)	51 (8.0)	15 (3.6)
Ct for etiologic detections, median (IQR)	25.0 (13.9–28.8)	16.4 (14.5–19.3)	21.2 (19.2–22.9)	19.3 (21.6–17.6)	24.0 (22.5–25.4)	23.6 (20.0–27.8)	20.8 (18.8–22.9)	23.8 (20.9–26.4)	19.4 (17.5–21.5)

Data are presented as no. (%) unless otherwise specified.

Abbreviations: Ct, cycle threshold; GII, genogroup II; IQR, interquartile range; SD, standard deviation; ST-ETEC, heat-stabile toxin-producing enterotoxigenic *Escherichia coli*.

**Figure 1.**
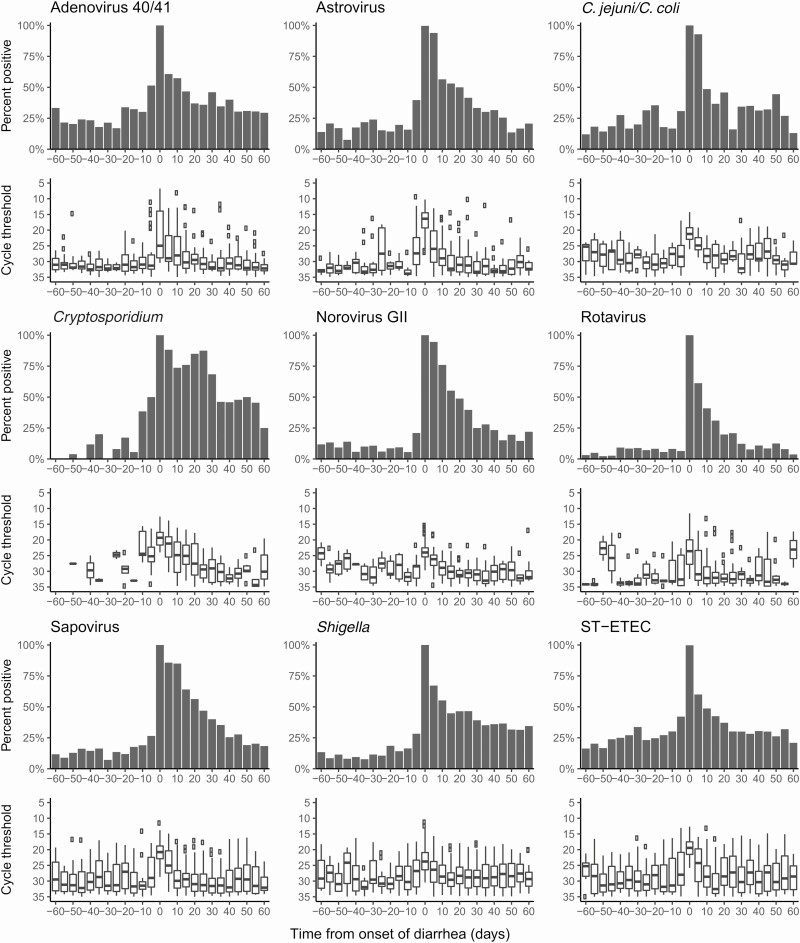
Proportion of stools positive (top) and box and whisker plots of the cycle threshold distribution of pathogen detections (bottom) for the top 9 causes of diarrhea in the Etiology, Risk Factors, and Interactions of Enteric Infections and Malnutrition and the Consequences for Child Health and Development (MAL-ED) cohort study in stools collected from 5-day intervals during the incubation (prediarrheal) and shedding (postdiarrheal) windows. Day 0 is the first day of study-defined diarrhea identified by active surveillance, thus, the proportion is 100% by definition. Abbreviations: C. jejuni/C. coli, Campylobacter jejuni/Campylobacter coli; GII, genogroup II; ST-ETEC, heat-stabile toxin-producing enterotoxigenic *Escherichia coli.*

First, we examined the prevalence and detection quantity of etiologic pathogens before and after each etiologic episode (Figure 1). There was a clearly asymmetric higher prevalence of etiologic pathogens in the shedding window compared to the incubation window and a declining prevalence and quantity of detection over the course of the shedding window. Pathogen prevalence in nondiarrheal stools in the 30 days prior to the onset of diarrhea ranged from <5% for rotavirus to approximately 25% for adenovirus 40/41, *Campylobacter jejuni/Campylobacter coli*, and ST-ETEC. For all etiologies, the etiologic pathogen was re-detected from the majority of stool samples obtained within 5 days after the onset of diarrhea.

**Figure 2.**
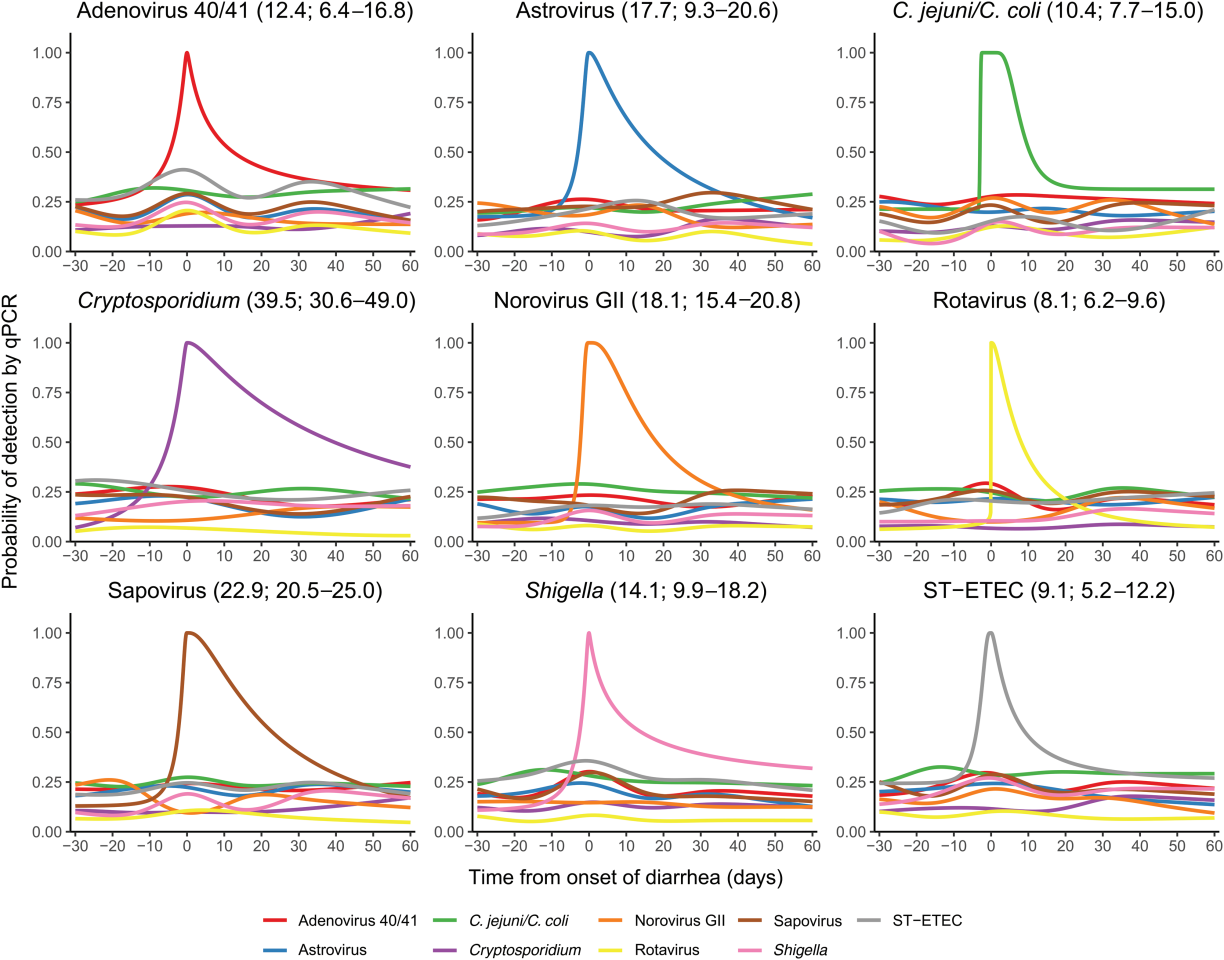
Estimated probability of pathogen detection from 30 days prior to the onset of diarrhea until 60 days after the onset of diarrhea for episodes of etiologic-specific diarrhea for each pathogen. The median duration of carriage and 95% confidence intervals are shown in parentheses after each pathogen. Abbreviations: C. jejuni/C. coli, Campylobacter jejuni/Campylobacter coli; GII, genogroup II; qPCR, quantitative polymerase chain reaction; ST-ETEC, heat-stabile toxin-producing enterotoxigenic *Escherichia coli.*

Among etiologic episodes for each pathogen, we then modeled the probability of the detection of all pathogens during the incubation and shedding windows (Figure 2). The remaining 8 nonetiologic pathogens served as negative controls for each model, with no clear evidence of an increased probability of detection of nonetiologic pathogens before, during, or after the etiologic episode, and no obvious trends in pathogen detection probability across the interval. The median duration of carriage after diarrhea onset varied from 8.1 (95% CI, 6.2–9.6) days for rotavirus to 39.5 (95% CI, 30.6–49.0) days for *Cryptosporidium* (Figure 2). Other pathogens with a median duration of carriage >14 days were astrovirus (17.7 [9.3–20.6] days), norovirus GII (18.1 [15.4–20.8] days), sapovirus (22.9 [20.5–25.0] days), and *Shigella* (14.1 [9.9–18.2] days). The results of the sensitivity analysis, which included all diarrheal episodes, were very similar with the exception of adenovirus 40/41, where the median duration of carriage increased from 9.2 (95% CI, 1.0–14.2) days when excluding repeated episodes within 60 days or without an intervening negative test to 12.4 (95% CI, 6.4–16.8) days when all episodes were included; no other estimates changed by >1.4 days.

Because it was possible that despite a shorter median duration of carriage, a subset of children could have prolonged shedding, we also estimated the difference in pathogen carriage from baseline (30 days before the onset of diarrhea) to 60 days after the onset of diarrhea (Table 2). The prevalence of pathogen carriage 60 days after the onset of diarrhea was at least 10% higher than the prevalence of pathogen carriage 30 days before the onset of diarrhea for *Cryptosporidium* (0.30 prevalence difference [95% CI, .23–.39]), *Shigella* (0.21 [95% CI, .16–.27]), and *C. jejuni/C. coli* (0.10 [95% CI, −.01 to .19]).

**Table 2.** Difference in the Model-predicted Probability of Detection of the Etiologic Pathogen 60 Days After Compared to 30 Days Prior to Diarrhea Onset

Pathogen	Prevalence Difference (95% CI)
Adenovirus 40/41	0.07 (.01–.15)
Astrovirus	−0.00 (−.05 to .12)
*Campylobacter jejuni/Campylobacter coli*	0.10 (−.01 to .19)
*Cryptosporidium*	0.30 (.23–.39)
Norovirus GII	0.06 (−.00 to .15)
Rotavirus	0.01 (−.02 to .04)
Sapovirus	0.04 (.00–.10)
*Shigella*	0.21 (.16–.27)
ST-ETEC	0.03 (−.04 to .08)

Abbreviations: CI, confidence interval; GII, genogroup II; ST-ETEC, heat-stabile toxin-producing enterotoxigenic *Escherichia coli*.

**Figure 3.**
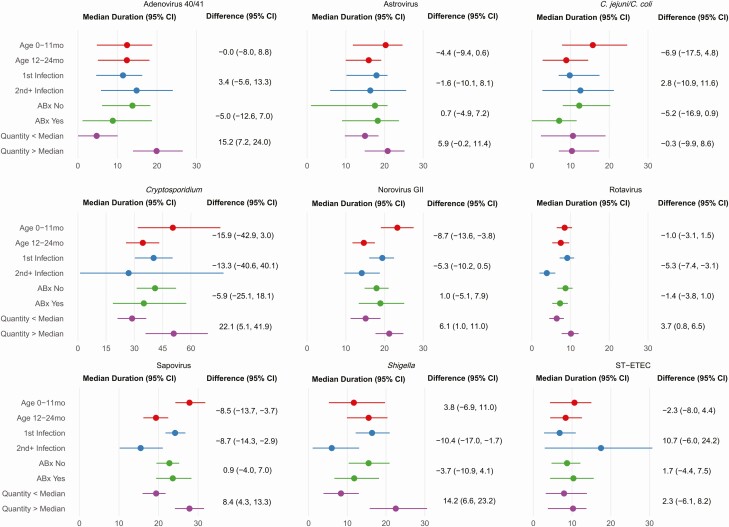
Estimated median duration of carriage stratified by age, infection order, use of an appropriate antibiotic, and pathogen quantity during diarrhea. Absolute differences between the estimates for each stratum are also shown. Abbreviations: ABx, antibiotic; CI, confidence interval; GII, genogroup II; ST-ETEC, heat-stabile toxin-producing enterotoxigenic *Escherichia coli.*

To interrogate factors associated with the duration of carriage, we estimated the median durations of carriage stratified by age in years, first or repeat etiologic episode for each child, treatment with appropriate empiric antibiotics, and the quantity of the etiologic pathogen in the diarrheal stool (Figure 3). There was evidence of a reduced duration of carriage in the second year of life for norovirus GII (−8.7 [95% CI, −13.6 to −3.8] days) and sapovirus (−8.5 [95% CI, −13.7 to −3.7] days), as well as with subsequent diarrhea episodes of norovirus GII (−5.3 [95% CI, −10.2 to .5] days), sapovirus (−8.7 [95% CI, −14.3 to −2.9] days), rotavirus (−5.3 [95% CI, −7.4 to −3.1] days), and *Shigella* (−10.4 [95% CI, −17.0 to −1.7] days). There was a small but non–statistically significant reduction in the duration of carriage of *Shigella* and *C. jejuni/C. coli* for episodes treated with appropriate empiric antibiotics. As expected, the quantity of pathogen detected during diarrhea was positively associated with the duration of postdiarrheal carriage.

Because of the temporal symmetry of both the available data (ie, monthly nondiarrheal samples were collected both before and after each etiologic episode), we also estimated the median duration of infection prior to the onset of diarrhea, that is, the incubation period (Table 3). As expected, the median probabilities of pathogen infection prior to each etiologic episode were substantially shorter. While the median duration of prediarrheal infection was 3 days or fewer for most pathogens, we found *Cryptosporidium* to have the longest duration of infection prior to the onset of diarrhea, with a median duration of almost 5 days, followed by adenovirus 40/41 and ST-ETEC.

**Table 3.** Median Duration of Detection of the Etiologic Pathogen Prior to Diarrhea Onset

Pathogen	Median Prediarrheal Detection, d (95% CI)
Adenovirus 40/41	4.7 (2.4–6.4)
Astrovirus	3.0 (.4–4.4)
*Campylobacter jejuni/Campylobacter coli*	3.0 (.2–3.7)
*Cryptosporidium*	4.9 (2.1–7.8)
Norovirus GII	2.3 (.8–3.3)
Rotavirus	0.0 (.0–2.7)
Sapovirus	2.4 (.3–3.6)
*Shigella*	2.5 (.6–3.8)
ST-ETEC	4.0 (.7–5.0)

Abbreviations: CI, confidence interval; GII, genogroup II; ST-ETEC, heat-stabile toxin-producing enterotoxigenic *Escherichia coli*.

## Discussion

Using a large, multisite birth cohort study, with intensive surveillance for incident diarrhea and regular collection of diarrheal and nondiarrheal stools tested with quantitative molecular diagnostics, we were able to systematically estimate the duration of postdiarrheal shedding of common enteropathogens among children in low-resource settings. These data provide a reference for the duration of shedding in these high-morbidity populations. There was a wide variation in the median duration of postdiarrheal enteropathogen shedding, ranging from approximately 1 week to >1 month. Many of the estimates are consistent with expectations—for example, the long duration of postdiarrheal carriage of *Cryptosporidium* and the caliciviruses and the longer incubation periods for *Cryptosporidium* and adenovirus 40/41 [6, 8, 9, 20, 21]. It is also notable that 3 diarrheal enteropathogens strongly implicated in child growth shortfalls, namely *Cryptosporidium*, *Campylobacter*, and *Shigella*, were most clearly associated with persistent shedding in this analysis [4, 22].

The long median durations of both pre-and postdiarrheal shedding of *Cryptosporidium* is consistent with historical studies using microscopy to identify oocyst excretion, in which the incubation period was 1 week, the duration of illness was close to 2 weeks, oocyst excretion continued for approximately 1 week after illness cessation, and a subset of patients had months of ongoing oocyst excretion [20]. The duration of shedding would be expected to be prolonged in immunocompromised individuals, including children with malnutrition [23]. A number of novel treatment strategies for *Cryptosporidium* have been identified, which, in addition to reducing the morbidity of an episode of cryptosporidiosis, may also help reduce the substantial reservoir of postdiarrheal shedding [24] and in turn the high observed rate of household transmission [25].

The duration of postdiarrheal shedding for *C. jejuni/C. coli* was relatively short, considering the strikingly high rates of subclinical carriage with this pathogen [26]. This suggests that the high subclinical prevalence is driven by (1) a high force of infection, much of which is not associated with clinical disease [27], and/or (2) chronic carriage in a subset of children, a described phenomenon that is thought to be facilitated by host adaptation [28, 29]. Although the estimate was imprecise, the high prevalence difference for pathogen carriage at the end of the shedding window in comparison to baseline, a difference that was out of proportion to the observed median duration of carriage, could be consistent with chronic carriage in a subset of children. However, it is difficult to estimate the relative contribution of this chronic carriage to the total burden of subclinical infection, and the force of infection is also likely high in these settings.

Similarly, we found that, despite a relatively short median duration of shedding, approximately 1 in 5 children had persistent *Shigella* shedding 2 months after the onset of diarrhea. Persistent clinical shigellosis has been described, as well as long-term asymptomatic carrier states [11, 30]. A number of characteristics of the pathogen may facilitate this state, including its ability to evade and subvert the immune response during intracellular growth [31]. Our analysis suggests that persistent subclinical carriage with *Shigella* occurs in a sizeable minority of children in these settings. This highlights the need for control strategies, whether appropriate antibiotic therapy or vaccines, that could help reduce pathogen transmission. *Shigella* vaccine development is now primarily focused on subunit vaccines that would be introduced later in the first year of life, such that indirect protection from reduced transmission may be critical for reducing the disease burden in infants [32]. The findings that the duration of postdiarrheal shedding was reduced by appropriate empiric antibiotic therapy as well as in nonprimary episodes of shigellosis, suggesting an impact of natural immunity, support the potential efficacy of such strategies.

The long duration of postdiarrheal shedding for these pathogens also has implications for the design and analysis of etiologic studies of diarrhea, in particular the impact of the definition of a nondiarrheal control and the incorporation of pathogen quantity [33]. We have previously demonstrated that the progressive restriction of nondiarrheal controls to those 7, 14, and 28 days remote from diarrhea increases the attribution for some pathogens, most notably for *Cryptosporidium*, norovirus, and sapovirus, the 3 pathogens with the longest median durations of postdiarrheal carriage in the present analysis [2]. The more stringent definition presumably reduces the detection of the etiologic pathogen in the control stools, increasing pathogen attribution when detected in diarrhea. Interestingly, however, these restrictions did not change estimates of *Shigella* diarrhea burden, suggesting that persistent *Shigella* carriage occurs at a sufficiently low quantity to not obscure the inference to etiology when detected in higher quantities in diarrheal stools. This supports the value of the incorporation of pathogen quantification for etiologic studies of diarrhea in settings with high carriage [1, 2].

Similar to estimates of postdiarrheal shedding, prior estimates of pathogen incubation periods come from heterogeneous sources. The long duration of prediarrheal carriage identified for *Cryptosporidium* in this study is consistent with a volunteer study of adults in which the median incubation period was 1 week [20]. It is also one of the only enteric pathogens for which presymptomatic shedding has been described [34]. Among viral etiologies, adenovirus 40/41 diarrhea had the longest prediarrheal duration of infection, which is consistent with a report of an outbreak among young children in the United Kingdom, where the median incubation period was 8–10 days [21]. Meanwhile, the median duration of infection of heat-stabile toxin-producing *E. coli* of 4 days is longer than is typically described in natural infection [35] or challenge studies in adults [36]. The short duration of infection prior to diarrhea onset for most pathogens is consistent with expectations that infections causing diarrhea represent new exposures and also supports the validity of the estimates of postdiarrheal durations of carriage predicted by the same modeling approach.

The estimates in this study come from the modeled probability of pathogen detection, taking advantage of the random intervals between diarrhea onset and prior and subsequent stool collections. Because we had relatively few postdiarrheal stools available for each episode, we could not estimate the duration of shedding for each episode and thus explicitly describe postdiarrheal carriage for each diarrhea episode. This would require more frequent postdiarrheal stool collection, which is unlikely to be available from a single study across a wide range of etiologies. Second, detection of enteric pathogen nucleic acid by qPCR cannot distinguish viable pathogen shedding. Thus, if used to infer the duration of shedding that can be directly implicated in pathogen transmission, these results are likely overestimates. However, we would presume that the relative duration of shedding of viable pathogens would be consistent with our findings here. Finally, we could not clearly distinguish postdiarrheal shedding from repeat exposure and infection during the shedding window. However, the clear asymmetry between pre- and postdiarrheal pathogen detection, the specificity of re-detection to the etiologic pathogen, and the consistency of the findings with prior single-pathogen studies all support that many of the postdiarrheal detections represent ongoing shedding after the diarrhea episode.

In summary, we were able to estimate the duration of postdiarrheal carriage of a broad range of enteropathogens. In the setting of strikingly high rates of subclinical carriage for some pathogens, clinical disease still represents a sentinel acquisition event that is associated with a substantial pathogen reservoir. Interventions targeted to identify and treat these episodes may help reduce transmission and other sequelae of subclinical infection.

## Notes

***Acknowledgments.*** The Etiology, Risk Factors and Interactions of Enteric Infections and Malnutrition and the Consequences for Child Health and Development Project (MAL-ED) is a collaborative project supported by the Bill & Melinda Gates Foundation, the Foundation for the National Institutes of Health (NIH), the NIH, and the Fogarty International Center. The authors thank the staff and participants of the MAL-ED Network Project for their important contributions.

***Financial support.*** This work was supported by the Bill & Melinda Gates Foundation (award numbers OPP1131125 and OPP1131114); and the NIH (grant numbers K23 AI114888 to J. P. M. and K01AI130326 to E. T. R. M.).

***Potential conflicts of interest.*** N. P. reports advisory board fees from GlaxoSmithKline and personal fees from Aspen Pharmacare, outside the submitted work. All other authors report no potential conflicts of interest. All authors have submitted the ICMJE Form for Disclosure of Potential Conflicts of Interest. Conflicts that the editors consider relevant to the content of the manuscript have been disclosed.
